# Effect of Continuous Positive Airway Pressure on Airway Inflammation and Oxidative Stress in Patients with Obstructive Sleep Apnea

**DOI:** 10.1155/2016/3107324

**Published:** 2016-06-30

**Authors:** Promsrisuk Tichanon, Khrisanapant Wilaiwan, Santamit Sopida, Pasurivong Orapin, Boonsawat Watchara, Intarapoka Banjamas

**Affiliations:** ^1^Department of Physiology, Faculty of Medicine, Khon Kaen University, Khon Kaen 40002, Thailand; ^2^Department of Medicine, Faculty of Medicine, Khon Kaen University, Khon Kaen 40002, Thailand; ^3^Bumrungrad International Hospital, Bangkok 10110, Thailand

## Abstract

*Background*. Airway inflammation and oxidative stress may be linked in obstructive sleep apnea (OSA) patients. We determined the effectiveness of continuous positive airway pressure (CPAP) therapy in reducing fractional exhaled nitric oxide (FeNO) and malondialdehyde (MDA) levels in OSA patients.* Methods*. Thirteen patients with OSA and 13 normal controls were recruited. FeNO and MDA levels were measured in the controls and in OSA patients before and after three months of CPAP therapy.* Results*. FeNO and MDA levels were higher in the patients compared to the age and gender matched controls (FeNO: 25.9 ± 5.0 versus 17.5 ± 5.9 ppb, *P* < 0.001; MDA: 14.6 ± 7.8 versus 2.1 ± 0.3 *μ*mol/L, *P* < 0.001). FeNO and MDA levels were lower post-CPAP compared to pre-CPAP (FeNO: 25.9 ± 5.0 versus 17.0 ± 2.3 ppb, *P* < 0.001; MDA: 14.6 ± 7.8 versus 10.0 ± 6.4 *μ*mol/L, *P* < 0.01). Apnea-hypopnea index (15.9 ± 6.6 versus 4.1 ± 2.1/h, *P* < 0.001) and mean arterial pressure (*P* < 0.01) decreased following CPAP treatment. Daytime mean SpO_2_ (*P* < 0.05) increased.* Conclusion*. Our study demonstrates that CPAP therapy yields clinical benefits by reducing upper airway inflammation and oxidative stress in OSA patients.

## 1. Introduction

Obstructive sleep apnea (OSA) is characterized by transient obstruction of the upper airway during sleep. It has been hypothesized that airway and systemic inflammation is associated with the obstruction of the upper airway in OSA [1]. Several studies have reported increased inflammatory and oxidative stress markers in OSA patients compared to control group [2, 3]. Airway inflammation in OSA is probably due to release of inflammatory cytokines and oxygen free radicals caused by intermittent nocturnal hypoxemia during episodes of apnea. Snoring-related mechanical trauma may also cause airway inflammation [1]. Reactive metabolites of nitric oxide (NO) may induce oxidation and nitration leading to tissue damage.

Airway inflammation can be assessed by measuring the levels of fractional exhaled nitric oxide (FeNO) [1]. Prior studies evaluating the FeNO levels in OSA were ambiguous. Various studies have shown that FeNO levels in OSA patients are reduced [4], increased [1], or no different [5] compared to control groups.

Ischemia-reperfusion injury caused by intermittent nocturnal hypoxemia produces oxygen free radicals [3]. Previous studies on this are also ambiguous, showing either a significant increase in oxidative stress [6] or no significant increase [7] in OSA patients.

Continuous positive airway pressure (CPAP) is considered the gold standard treatment for OSA patients. Extensive CPAP use results in significant clinical benefits, including decreases in airway inflammation, oxidative stress, and cardiovascular risk [8]. The effect of CPAP on FeNO and oxidative stress biomarkers is unclear. In previous studies, FeNO levels were significantly lowered following CPAP treatment [9, 10]. In contrast, development of airway hyperresponsiveness was observed after CPAP therapy, suggesting increased airway inflammation [11]. Most previous studies show a significant decrease in oxidative stress during CPAP treatment [12, 13]. However, one study found no evidence of increased MDA levels (a marker of oxidative stress) in patients whose moderate-to-severe OSA returned after stopping CPAP therapy [14]. Additionally, previous studies have demonstrated that CPAP treatment results in either a reduction [13] or no change [15] in daytime sleepiness in patients with OSA. We therefore investigated whether FeNO and oxidative stress levels were elevated in OSA and evaluated the effectiveness of the CPAP therapy in reducing oxidative stress, decreasing daytime sleepiness, and improving airway inflammation in Thai OSA patients.

## 2. Methods

### 2.1. Study Subjects

Twenty-four patients whose ages were between 30 and 70 years volunteered to participate in the study. Patients were recruited between October 2014 and February 2015. The non-OSA group was age and gender matched and consisted of healthy 10 male and 3 female subjects with no history of OSA or other diseases. Patients were treated with CPAP while sleeping at least five hours per night for at least five days per week for three consecutive months. Patients were newly diagnosed with OSA by medical specialists using polysomnography (PSG) within the month preceding the start of the study. Patients included in this study had an apnea-hypopnea index (AHI) of at least five per hour, no history of treatment for OSA, and no history of cardiovascular disease (e.g., coronary heart disease and myocardial infarction). Patients with a history of autoimmune conditions or symptoms of respiratory tract infection in the six weeks prior to the study and history of sleep apnea treatment with CPAP or oral devices, tracheostomy, or use of oxygen therapy at home were excluded. Study subjects with history of other diseases associated with high FeNO (e.g., viral respiratory tract infections, systemic lupus erythematosus, liver cirrhosis, acute lung allograft rejection, and posttransplant bronchiolitis obliterans) or history of other diseases associated with low FeNO (i.e., cystic fibrosis, HIV infection, and pulmonary hypertension) were excluded as well. The Thai Clinical Trials Registry (TCTR) identification number of this study is TCTR20160413001.

### 2.2. CPAP Therapy and Follow-Up

CPAP (DeVilbiss IntelliPAP AutoAdjust, USA) was administered during sleep at night for at least five hours per night, for at least five days per week for three consecutive months. After one, two, and three months of CPAP therapy, the patients came for a follow-up visit during which average hours of nightly use (h) and average days per week (days/week) of CPAP therapy for each patient were recorded from the CPAP device.

### 2.3. Polysomnography (PSG)

PSG was performed according to the American Academy of Sleep Medicine (AASM) guidelines [16]. All patients underwent full-night PSG using a digital system at the Sleep Disorder Clinics, Faculty of Medicine (Srinagarind Hospital, Khon Kaen University). The parameters recommended by the AASM were monitored simultaneously and continuously. Thermocouple and nasal pressure were used for airflow detection, electroencephalography (EEG) tracings for assessing the total sleep time, bilateral electrooculography (EOG) and electrocardiography (ECG) for measuring heart rate, and anterior tibialis electromyography (EMG) to detect movement and arousals and to assess periodic limb movements. Inductance, oxyhemoglobin saturation, body position, and the presence or absence of snoring were recorded to assess thoracic and abdominal respiratory effort. Apnea-hypopnea index (AHI), AI, and CPAP pressure following one, two, and three months of CPAP therapy were also measured. Obstructive apnea was defined as a decrease in amplitude of airflow of at least 90% for at least 10 seconds and continued respiratory effort. Similarly, hypopnea was defined as a reduction in airflow of at least 30% that coincided with a decrease in oxygen desaturation of at least 3% [17].

### 2.4. Assessment of FeNO Levels

FeNO was measured with the Quark NO breath (COSMED Srl, ITALY) with a single-breath online method at constant flow of 50 mL/s or 12 seconds of exhalation of adults according to American Thoracic Society/European Respiratory Society guidelines, with a sensitivity of one part per billion (ppb) [18]. Calibration of the analyzer was automatically performed by the software. Briefly, after inhaling to total lung capacity, the subjects exhaled through a mouthpiece (NObreathFloTM mouthpiece or bacteria filter) into an exhalation circuit. All subjects were asked to refrain from eating, drinking, and strenuous exercise for two hours prior to FeNO measurement.

### 2.5. Assessment of MDA Levels

The level of MDA was measured following previously described methods [19]. MDA reacts with thiobarbituric acid in boiling water to form a colored complex called thiobarbituric acid-reactive substance (TBARS), which can be detected by a spectrophotometric assay. Plasma samples (150 *µ*L) were treated with 10% TCA, 5 mM EDTA, 8% SDS, and 0.6 *µ*g/mL of BHT. The mixture was incubated for 10 minutes at room temperature, and then 0.6% TBA was added, and the mixture was boiled in a water bath for 30 minutes. After cooling to room temperature, the mixture was centrifuged at 10,000 ×g for five minutes. The absorbance of the supernatant was measured at 532 nm by a spectrophotometer. A standard curve was generated with appropriate concentrations of 1,1.3,3-tetraethoxypropane (0.3–10 *µ*M).

### 2.6. Statistical Analyses

Demographic, anthropometric, and PSG data were analyzed. Most data were expressed as mean ± SD. A Shapiro-Wilk *W*-test was used to examine normal distribution of data. A paired *t*-test was used to compare demographic and anthropometric data, FeNO, and MDA before and after the three consecutive months of CPAP therapy. The two-sample Wilcoxon rank-sum (Mann-Whitney) test was used to compare nonparametric data. An analysis of covariance (ANCOVA) was used to compare FeNO levels between OSA patients and controls with an adjusted body mass index. Statistical analyses were made using STATA version 12.0 (Stata Corp., College Station, TX). A value of *P* < 0.05 was considered statistically significant.

## 3. Results

**Figure 1:**
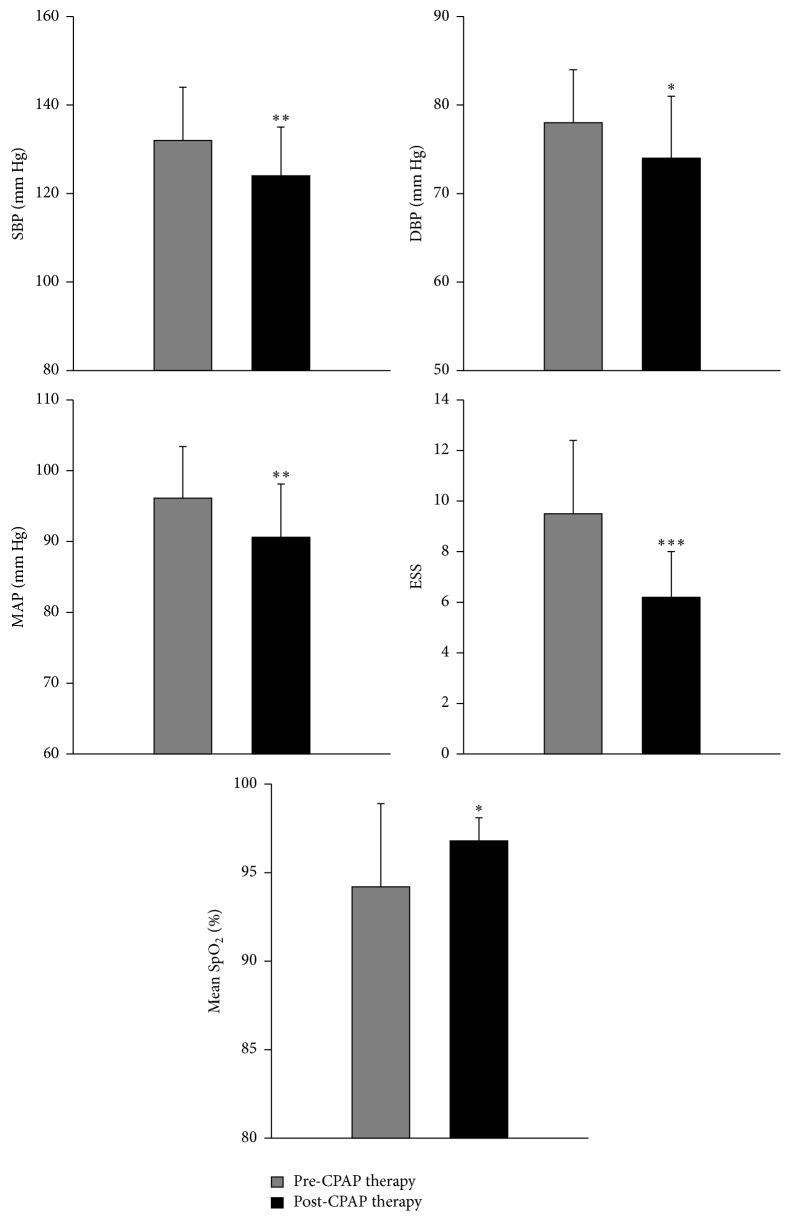
Systolic blood pressure (SBP), diastolic blood pressure (DBP), mean arterial pressure (MAP), Epworth Sleepiness Scale (ESS), and mean oxygen saturation (mean SpO_2_) in 13 obstructive sleep apnea (OSA) patients before and after continuous positive airway pressure (CPAP) therapy. ^*∗*^ *P* < 0.05, ^*∗∗*^ *P* < 0.01, and ^*∗∗∗*^ *P* < 0.001 pre- versus post-CPAP therapy.

The 24 patients were initially recruited to the study based only on their score on the Epworth Sleepiness Scale. After recruitment, each patient's OSA diagnosis was confirmed by polysomnography, and it turned out that eight of them had AHI of ≤5/h. These eight were excluded from the study. The remaining 16 patients were studied but three patients dropped out due to lack of CPAP compliance. Ten men and three women completed the study.  Table 1 presents demographic data before and after CPAP therapy. The male-to female patient ratio was 3.3. Most of the patients were in their fifties. All OSA patients in this study were obese. Nine had controlled hypertension while four were normotensive. After three consecutive months of CPAP therapy, patients exhibited significantly decreased diastolic blood pressure (DBP) (*P* < 0.05), systolic blood pressure (SBP) (*P* < 0.01), MAP (*P* < 0.01), and Epworth Sleepiness Scale (ESS) (*P* < 0.001). In addition, improved daytime mean SpO_2_ (*P* < 0.05) was also observed (Table 1 and Figure 1). BMI, neck circumference (NC), waist circumference (WC), hip circumference (HC), waist-to-hip ratio (WHR), and heart rate (HR) at rest were not altered by CPAP therapy (Table 1).

**Figure 2:**
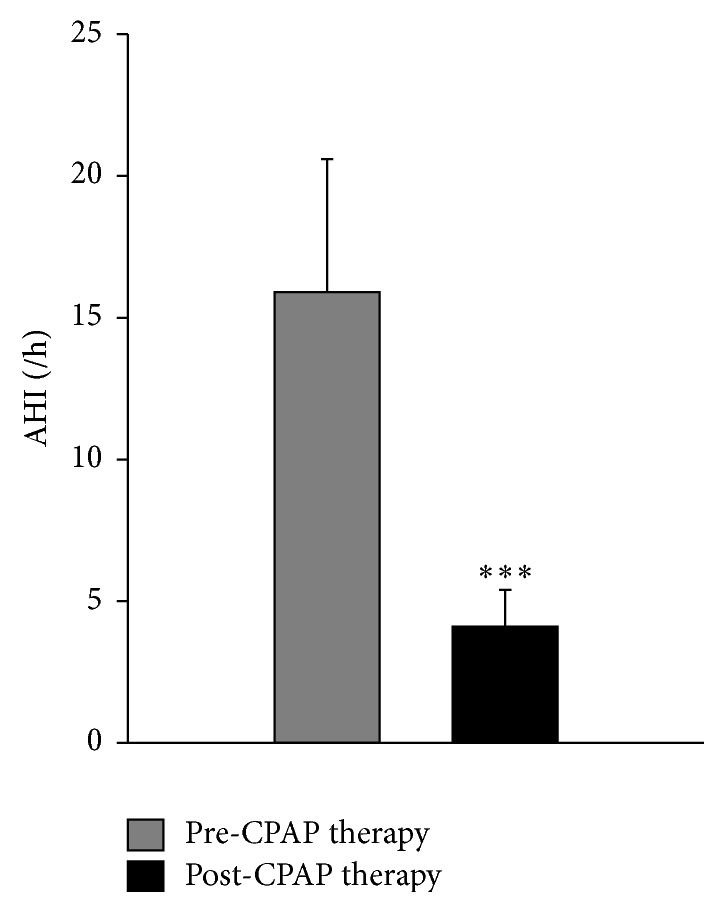
Effect of continuous positive airway pressure (CPAP) therapy on apnea-hypopnea index (AHI) in 13 obstructive sleep apnea (OSA) patients. ^*∗∗∗*^ *P* < 0.001 pre- versus post-CPAP therapy.

PSG data measured in OSA patients before CPAP therapy are shown in Table 2 and Figure 2. The mean AHI was 15.9 ± 6.6/h of total sleep time (TST). Six patients had an AHI of 6.4 to 13.0/h, six had AHI of 15.6 to 21.9/h, and one had AHI of 31.4/h. The AI of 36.8 ± 9.9/h ranged from 16.9 to 47.4/h. Mean sleep efficiency was reduced to 78.9 ± 13.7% compared to the normal value of 90%. The average CPAP use was 6.4 ± 0.9 hours per night and 5.4 ± 0.4 days per week. There was no difference in CPAP pressure used during pre- and post-CPAP treatment. AHI gradually decreased over time following CPAP treatment. After three consecutive months of CPAP therapy, AHI as measured by the autoPAP device was significantly reduced to 4.1  ±  2.1/h compared to the pre-CPAP value of 15.9 ± 6.6/h (*P* < 0.001).

**Figure 3:**
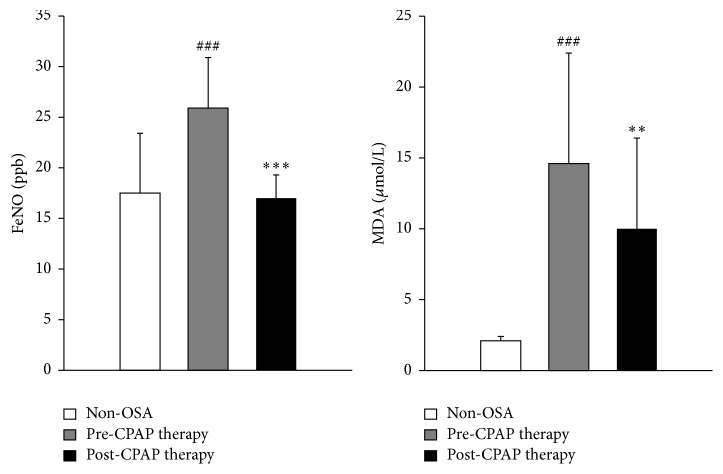
Effect of continuous positive airway pressure (CPAP) therapy on fractional exhaled nitric oxide (FeNO) and malondialdehyde (MDA) levels in 13 obstructive sleep apnea (OSA) patients and 13 non-OSA controls. ^*∗∗*^ *P* < 0.01 and ^*∗∗∗*^ *P* < 0.001 pre- versus post-CPAP therapy, ^###^ *P* < 0.001 non-OSA versus pre-OSA therapy.

FeNO levels in patients with OSA showed a significant decrease of 34.4% following CPAP therapy compared to pre-CPAP therapy levels (*P* < 0.001). FeNO values ranged from 17.0 to 34.5 ppb before CPAP therapy and 12.5 to 21.5 ppb after CPAP therapy. Moreover, FeNO levels were significantly higher in OSA patients than in controls (25.9 ± 5.0 versus 17.5 ± 5.9 ppb, *P* < 0.001) (Table 3 and Figure 3).

MDA levels in OSA patients were 6.9 times higher than those of the controls (14.6 ± 7.8 versus 2.1 ± 0.3 ppb; *P* < 0.001). MDA levels showed a significant decrease of 31.5% compared to pre-CPAP therapy (*P* < 0.01). OSA patients had ranges of MDA between 7.0 and 35.8 *μ*mol/L before CPAP and 5.3 and 29.7 *μ*mol/L after CPAP therapy (Table 3 and Figure 3).

## 4. Discussion

The present study demonstrates decreases in oxidative stress, airway inflammation, AHI, ESS, SBP, DBP, and MAP after three months of CPAP therapy in OSA patients. Moreover, significantly increased daytime mean SpO_2_ was also observed.

We observed significant effects on MAP after CPAP treatment. This observation is consistent with findings in previous studies that showed that treatment of severe OSA patients with CPAP for three and six months resulted in a significant decrease in arterial blood pressure [20, 21]. The reduced levels of MAP we observed were likely due to alleviation of endothelial dysfunction, since we also observed reduced levels of MDA.

Airway inflammation plays a key role in the pathogenesis of OSA. Airway inflammation in OSA patients may be caused by physical injury to the mucosal lining caused by repetitive airway closure and reopening [22]. Increased FeNO levels in OSA patients may reflect a systemic inflammatory response [1]. In this study, we observed that FeNO levels decreased following three months of CPAP in OSA patients compared to pre-CPAP. This observation is in agreement with recent studies showing a significant decrease in the FeNO levels following the CPAP therapy in OSA patients compared to a control group [9, 10]. FeNO concentrations normalized after three months of CPAP therapy, suggesting reduction of inflammation in endothelium in the OSA patients [4]. Four hours of CPAP use per night proved effective for impaired OSA patients in order to achieve normal scores in the ESS [23]. Longer therapy duration and better compliance might further decrease airway inflammation [24].

Oxidative stress from recurrent nocturnal oxygen desaturation may be present in OSA patients. The effect of CPAP on oxidative stress biomarkers is unclear. Males with severe OSA showed a significant increase in oxidative stress after 12 weeks of CPAP therapy (6.2 hours per night) compared to a control group [25]. The present study using CPAP for three months decreased MDA levels compared to pre-CPAP therapy. This finding is comparable to the majority of other studies that suggest that using CPAP for four hours or more per night significantly reduces MDA levels [6, 20, 26]. CPAP is effective in suppressing OSA-associated changes in redox status [27]. It reduces chronic intermittent hypoxia, which triggers production of oxidative stress [11]. The use of CPAP in OSA patients resulted in decreased oxidative stress levels and reduction or elimination of episodes of hypoxia.

Our study showed a significant decrease in daytime sleepiness after CPAP, which is in agreement with the majority of previous studies [13, 21]. Moreover, a previous study suggests that reduced ESS is strongly correlated with increased CPAP use [28]. Likewise, improvement in mean SpO_2_ at night after three months of treatment with CPAP has also been observed. It is likely that intermittent hypoxia found in OSA patients was reduced by CPAP treatment, as AHI was reduced from 15.9.1 to 4.1/h.

CPAP pressure of less than 20 cm H_2_O during sleep does not have any adverse effects on lung function in humans [29]. However, CPAP treatment might lead to reductions in cardiac output and blood pressure, especially in the presence of impaired right ventricular function [30]. On average, effective CPAP therapy requires pressure between 6 and 14 cm H_2_O. Individual pressure requirements were calculated for patients based on findings from a sleep study in our laboratory. The CPAP regimen used in the present study, 11.5  ±  1.5 cm H_2_O for three consecutive months, is similar to that used in previous studies [9, 12, 13].

## 5. Conclusions

The present study suggests clinical benefit of CPAP therapy of 6.4 ± 0.9 hours per night and 5.4 ± 0.4 days per week for three consecutive months in patients with mild to severe OSA. We observed increases in mean oxygen saturation and reduction in daytime sleepiness, arterial blood pressure, airway inflammation, and oxidative stress.

## Figures and Tables

**Table 1:** Demographic data in OSA patients pre- and post-CPAP therapy and non-OSA controls.

	Non-OSA (*N* = 13)	Pre-CPAP (*N* = 13)	Post-CPAP (*N* = 13)
Age (years)	52.5 ± 12.3	53.1 ± 12.4	—
Gender (M/F)	10/3	10/3	10/3
BMI (kg/m^2^)	23.3 ± 1.5	28.4 ± 3.5^##^	28.1 ± 3.8
Neck circumference (cm)	36.9 ± 3.4	41.4 ± 2.6^##^	41.5 ± 3.4
Waist circumference (cm)	83.2 ± 5.7	102.7 ± 7.5^###^	101.8 ± 7.3
Hip circumference (cm)	92.8 ± 2.9	107.5 ± 9.4^###^	107.6 ± 8.7
Waist-to-hip ratio	0.90 ± 0.05	0.96 ± 0.05^###^	0.95 ± 0.04
Mean oxygen saturation (%)	98.2 ± 0.4	94.2 ± 4.7^#^	96.8 ± 1.3^*∗*^
Epworth sleepiness scale	2.9 ± 1.2	9.5 ± 2.9^###^	6.2 ± 1.8^*∗∗∗*^
Heart rate at rest (/min)	77 ± 10	77 ± 11	72 ± 8
Systolic BP (mm Hg)	123 ± 15	132 ± 12	124 ± 11^*∗∗*^
Diastolic BP (mm Hg)	75 ± 10	78 ± 6	74 ± 7^*∗*^
Mean arterial pressure (mm Hg)	90.9 ± 11.0	96.1 ± 7.3	90.6 ± 7.5^*∗∗*^
Controlled hypertension (*n*)	0	9	9
Normotension (*n*)	13	4	4

Data are expressed as mean ± SD. OSA: obstructive sleep apnea; CPAP: continuous positive airway pressure; BMI: body mass index; BP: blood pressure. ^*∗*^ *P* < 0.05, ^*∗∗*^ *P* < 0.01, and ^*∗∗∗*^ *P* < 0.001 pre- versus post-CPAP therapy; ^#^ *P* < 0.05, ^##^ *P* < 0.01 and ^###^ *P* < 0.001 non-OSA versus pre-CPAP therapy.

**Table 2:** Polysomnographic data prior to CPAP therapy in OSA patients.

	OSA patients (*N* = 13)
Apnea index (/h)	5.7 ± 3.8
Hypopnea index (/h)	10.2 ± 6.0
Apnea hypopnea index (/h)	15.9 ± 6.6 (0 m), 4.1 ± 2.1 (3 m)^*∗∗∗*^
Sleep efficiency (%)	78.9 ± 13.7
Total sleep time (min)	315.0 ± 66.0
Time in bed (min)	401.0 ± 86.5
Lowest oxygen saturation (%)	64.9 ± 11.4
Arousal index (/h)	36.8 ± 9.9
CPAP average nightly use (h)	6.4 ± 0.9
CPAP average weekly use (day)	5.4 ± 0.4
CPAP pressure (cm H_2_O)	8.0 ± 0.5 (0 m), 11.5 ± 1.5 (3 m)

The data for apnea hypopnea index and CPAP pressure represent measurements pre- and post-CPAP therapy. OSA: obstructive sleep apnea; h: hour; CPAP: continuous positive airway pressure; 0 m: before CPAP; 3 m: 3 months after CPAP. ^*∗∗∗*^ *P* < 0.001 pre- versus post-CPAP therapy.

**Table 3:** FeNO and MDA levels before and after three months of CPAP therapy in OSA and in non-OSA patients.

	Non-OSA (*N* = 13)	Pre-CPAP (*N* = 13)	Post-CPAP (*N* = 13)
FeNO (ppb)	17.5 ± 5.9 (8.0–24.0)	25.9 ± 5.0^###^ (17.0–34.5)	17.0 ± 2.3^*∗∗∗*^ (12.5–21.5)
MDA (*μ*mol/L)	2.1 ± 0.3 (1.4–2.4)	14.6 ± 7.8^###^ (7.0–35.8)	10.0 ± 6.4^*∗∗*^ (5.3–29.7)

Data are presented as mean ± SD. FeNO: fractional exhaled nitric oxide; MDA: malondialdehyde; ppb: parts per billion. ^*∗∗*^ *P* < 0.01 and ^*∗∗∗*^ *P* < 0.001 pre- versus post-CPAP therapy; ^###^ *P* < 0.001 non-OSA versus pre-OSA.
